# Factors Affecting the Vitamin C Dose-Concentration Relationship: Implications for Global Vitamin C Dietary Recommendations

**DOI:** 10.3390/nu15071657

**Published:** 2023-03-29

**Authors:** Anitra C. Carr, Jens Lykkesfeldt

**Affiliations:** 1 Nutrition in Medicine Research Group, Department of Pathology and Biomedical Science, University of Otago, Christchurch 8011, New Zealand; 2 Faculty of Health & Medical Sciences, University of Copenhagen, DK-1870 Frederiksberg C, Denmark

**Keywords:** vitamin C, ascorbic acid, vitamin C requirements, vitamin C recommendations, body weight, obesity, smoking, aging, socioeconomic status, NHANES

## Abstract

Vitamin C status is known to be associated with several demographic and lifestyle factors. These include gender, age, ethnicity, pregnancy/lactation, body weight, smoking status and dietary habits. In the present study, our aim was to investigate the National Health and Nutrition Examination Survey (NHANES) 2017–2018 datasets to assess the impact of these factors on vitamin C dose-concentration relationships to establish if there are higher requirements for vitamin C in certain subpopulations, and the possible extent of these additional requirements. The final cohort comprised 2828 non-supplementing adult males and females (aged 18–80+ years) with both vitamin C serum concentrations and dietary intake data available. The data were subsequently stratified by gender, age tertiles (≤36, 37–58, ≥59 years), ethnicity (non-Hispanic white, non-Hispanic black, and total Hispanic), socioeconomic tertiles (poverty income ratios: ≤1.35, 1.36–3.0, >3.0), weight tertiles (<72, 72–91, >91 kg), BMI tertiles (<26, 26–32, >32 kg/m^2^) and smoking status. Sigmoidal (four parameter logistic) curves with asymmetrical 95% confidence intervals were fitted to the dose-concentration data. We found that males required vitamin C intakes ~1.2-fold higher than females to reach ‘adequate’ serum vitamin C concentrations of 50 µmol/L. Males had both higher body weight and a higher prevalence of smoking than females. Smokers required vitamin C intakes ~2.0-fold higher than non-smokers to reach adequate vitamin C concentrations. Relative to adults in the lighter weight tertile, adults in the heavier weight tertile required ~2.0-fold higher dietary intakes of vitamin C to reach adequate serum concentrations. We did not observe any impact of ethnicity or socioeconomic status on the vitamin C dose-concentration relationship, and although no significant difference between younger and older adults was observed at vitamin C intakes > 75 mg/day, at intakes < 75 mg/day, older adults had an attenuated serum response to vitamin C intake. In conclusion, certain demographic and lifestyle factors, specifically gender, smoking and body weight, have a significant impact on vitamin C requirements. Overall, the data indicate that the general population should consume ~110 mg/day of vitamin C to attain adequate serum concentrations, smokers require ~165 mg/day relative to non-smokers, and heavier people (100+ kg) require ~155 mg/day to reach comparable vitamin C concentrations. These findings have important implications for global vitamin C dietary recommendations.

## 1. Introduction

Vitamin C is a ubiquitous nutrient in plants and most animals, but is essential in humans due to the loss of the ability of our livers to synthesize the vitamin [1]. The vitamin has vital cofactor functions in numerous biosynthetic and regulatory pathways and is, thus, strongly implicated for optimal human health [2]. Assessment of global vitamin C status indicates that populations in high income countries generally have mean vitamin C concentrations of approximately 50 µmol/L [3]. This is also considered an ‘adequate’ circulating concentration by the European Food Safety Authority (EFSA) [4], and is attained by intakes between 60 and 100 mg/day in healthy men and women [5,6,7]. Due to vitamin C’s non-linear uptake kinetics, plasma concentrations generally saturate at approximately 70 µmol/L, which occurs at intakes between 200 and 400 mg/day [5,6,7]. Plasma concentrations of ≤23 µmol/L are considered hypovitaminosis C, and those ≤11 µmol/L are considered deficient and at increased risk of developing the potentially fatal deficiency disease scurvy [8].

A number of demographic and lifestyle factors have been shown to impact circulating vitamin C concentrations and, thus, potentially increase the intake requirements for the vitamin [9]. These include gender, age, ethnicity, socioeconomic status, pregnancy/lactation, weight/BMI and smoking. It is well established that gender has an impact on vitamin C status, with males generally presenting with lower vitamin C status than females, even when consuming comparable or higher dietary intakes of the vitamin [3]. The effect of age on vitamin C requirements is less clear. Many studies have shown either no change or a decrease in vitamin C status in non-supplementing older people [10,11]. However, we recently reported that older people with lower vitamin C intakes may be at higher risk of insufficiency than younger people with similar intakes [12]. Previous studies have reported varying vitamin C status amongst different ethnicities and those from different socioeconomic status, which has been attributed to differing dietary, environmental and lifestyle factors [9]. Weight is known to affect the vitamin C dose-concentration relationship through a volumetric dilution mechanism, whereby the same dose distributed in a larger volume becomes more dilute [13]. This is one of the reasons why males tend to have lower vitamin C status than females, despite comparable or even higher dietary intakes [14]. Volumetric dilution may also contribute to decreasing vitamin C status as pregnancies progress. Smokers are well known to have lower vitamin C status than non-smokers [9], due to both enhanced oxidative stress and a generally lower dietary intake of the vitamin [15,16]. Higher turnover and requirements of the vitamin in smokers have been demonstrated using radiolabel and matched dietary intake studies [17,18].

Globally, dietary intake recommendations for vitamin C vary widely, from 40 to 110 mg/day, with some countries recommending up to 200 mg/day for optimal health [19]. Some health authorities recommend lower intakes for females, generally extrapolated from their lower body weight compared to males [19]. Age and ethnicity are usually not taken into account, other than a slightly higher recommendation for older adults in France [4,20]. Smoking status is considered by a handful of authorities, with an additional 20–80 mg/day of the vitamin recommended for smokers [19]. To date, the impact of body weight on vitamin C status has not been taken into consideration by health authorities, despite the growing prevalence of obesity worldwide [21]. Nevertheless, the introduction of a higher body weight category is currently under consideration for vitamin C recommendations within the Nordic Nutrition Recommendations 2022 update [22].

In this study, we investigated the National Health and Nutrition Examination Survey (NHANES) 2017–2018 datasets to establish the impact of various demographic and lifestyle factors (i.e., gender, smoking, weight/BMI, age, ethnicity, and socioeconomic status) on the vitamin C dose-concentration relationship to establish if there are higher requirements for vitamin C in certain subpopulations and the potential extent of these requirements. Although both pregnancy and lactation are known to increase the requirements for vitamin C [19], dose-concentration relationships could not be determined due to the small number of pregnant and lactating volunteers in the NHANES cohort. The impact of various demographic and lifestyle factors on vitamin C intake requirements and the implications for future dietary recommendations is discussed.

## 2. Materials and Methods

### 2.1. The NHANES 2017–2018 Cohort

We used the NHANES 2017–2018 datasets for the present study [23]. These datasets comprise a nationally representative survey of the non-institutionalized civilian US population based on a multistage probability sample. All participants provided informed consent and all identifying information was removed prior to the datasets being made publicly available [23]. For the current analyses, inclusion criteria consisted of both sexes and all ethnicities, ages ≥ 18 years of age, of non-institutionalized civilian participants who were able to give informed consent, and who participated in both questionnaire and laboratory measurements (*n* = 7435). Exclusion criteria were: age < 18 years, missing serum vitamin C concentrations, missing day 1 and/or day 2 dietary intake data, and supplementing with vitamin C-containing supplements. The final cohort consisted of 2828 individuals.

### 2.2. Demographic Information

The following data were extracted from the NHANES datasets: sex (male or female), age (range 18 to 80+ years), ethnicity (non-Hispanic white, non-Hispanic black, Mexican American, other Hispanic, or other), ratio of family income to poverty (range 0 to 5+), weight and body mass index (BMI), smoking status (use of tobacco/nicotine in the last 5 days), and the number of cigarettes smoked per day was calculated.

### 2.3. Dietary Vitamin C Intakes

Dietary vitamin C intakes were estimated using the Dietary Data Questionnaire dataset acquired from the What We Eat In America Questionnaire developed by the USA Department of Agriculture and USA Department of Health and Human Services. Two days of 24-h dietary recall data were collected through an initial in-person interview in the mobile exam clinic, and a second interview was conducted over the telephone within 3 to 10 days. The USDA Food and Nutrient Database for Dietary Studies, 2.0 (FNDDS 2.0) was used to calculate the corresponding vitamin C intake. Mean intakes are presented as mg/day.

### 2.4. Serum Vitamin C Concentrations

Blood samples were collected from participants with a median fasting time of 10.5 (5.25–12.75) hours. The processed serum was immediately mixed with four parts 6% metaphosphoric acid, aliquoted and frozen at −70 °C. Vitamin C (ascorbic acid) was measured using isocratic ultra-high performance liquid chromatography (UPLC) with electrochemical detection [24]. Vitamin C concentrations are presented as µmol/L.

### 2.5. Data Analyses

Median and interquartile range (Q1, Q3) or mean and standard deviation (SD) were used for continuous variables and counts with percentages were used for categorical variables. Group differences were assessed using non-parametric Mann–Whitney U tests or Kruskal–Wallis tests with Dunn’s post-hoc test to correct for multiple comparisons. *p* values < 0.05 signified statistical significance. Linear regression correlations were determined using Spearman *r*. Sigmoidal (four parameter logistic) curves with asymmetrical 95% confidence intervals were fitted to dose-concentration data to estimate the vitamin C intakes required to reach ‘adequate’ serum vitamin C concentrations of 50 µmol/L and maximal serum concentrations attained at steady-state intakes of 200 mg/day. To calculate intake differences and relative requirements, the upper 95% CI of the first curve was related to the lower 95% CI of the second curve. Data analyses and graphical presentations were carried out using GraphPad Prism 9 (GraphPad, San Diego, CA, USA).

## 3. Results

### 3.1. Vitamin C Dose-Concentration Relationship of the Total Cohort

The total cohort comprised 2828 participants (Table 1). The age range was 18 to 80+ years with a median (Q1, Q3) age of 48 (32, 62) years. Of the total cohort, 50% were male and 25% had smoked within the previous 5 days. One third of the cohort were non-Hispanic white and one quarter non-Hispanic black. The median body weight of the cohort was 80 (68, 97) kg and the median BMI was 29 (25, 34) kg/m^2^. The median vitamin intake was 53 (24, 102) mg/day, which exhibited skewness, resulting in a mean intake of 75 (95% CI 72, 78). The vitamin C status of the cohort was 43 (23, 60) µmol/L, which was normally distributed.

**Figure 1:**
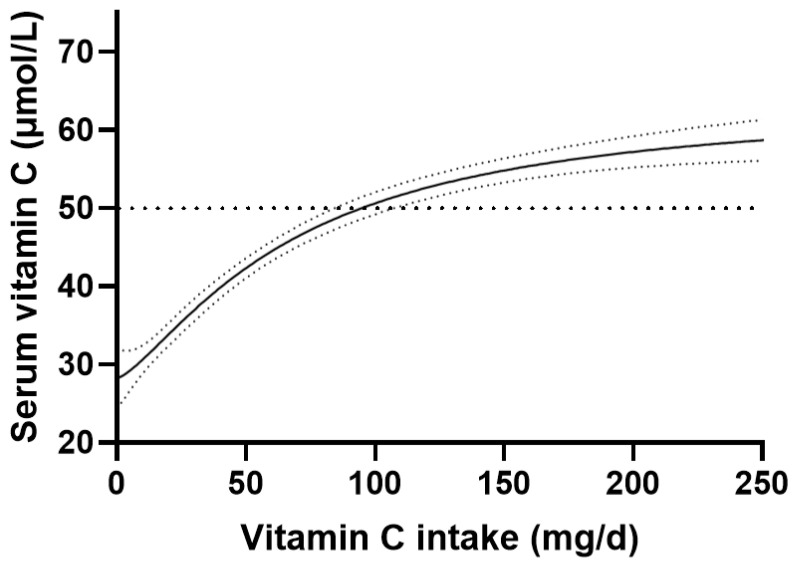
Vitamin C dose-concentration relationship of the total cohort (*n* = 2828). Sigmoidal (four parameter logistic) curve was fitted to the dose-concentration data with asymmetrical 95% confidence intervals indicated. Dashed line indicates 50 µmol/L serum vitamin C, which is considered ‘adequate’.

The vitamin C dose-concentration relationship of the total cohort is shown in Figure 1. This follows the characteristic sigmoidal relationship typically observed for vitamin C [5,6]. The cohort, as a whole, reached a serum concentration of 50 µmol/L (considered ‘adequate’) at a vitamin C dietary intake of 93 (83, 107) mg/day. The serum vitamin C concentration reached at a steady-state dietary intake of 200 mg/day was 57 (55, 59) µmol/L.

### 3.2. Vitamin C Dose-Concentration Relationship Relative to Gender

As previously reported in many observational studies, males had significantly lower vitamin C status (39 [21, 55] µmol/L) relative to females (47 [27, 64] µmol/L), despite comparable dietary intakes (Table 2). There were significant differences between the genders for body weight and BMI with males having higher body weight but lower BMI than females. There was also a higher prevalence of smoking for males relative to females (29% vs. 19%, respectively).

**Figure 2:**
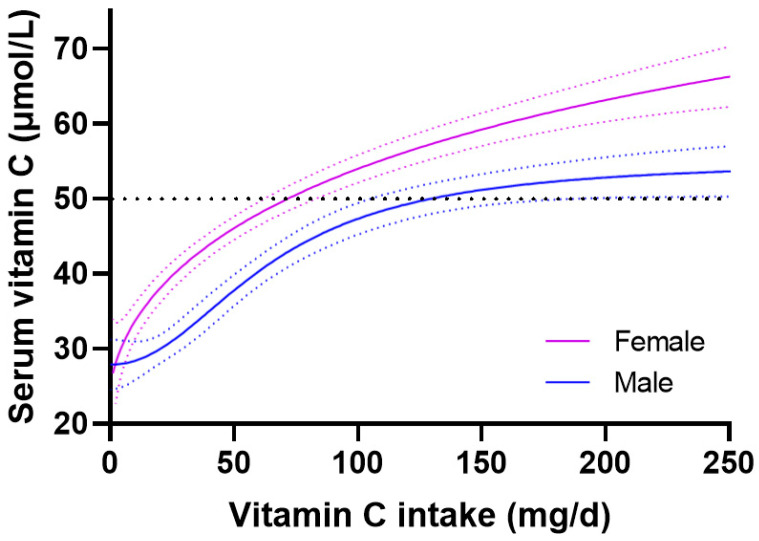
Vitamin C dose-concentration relationship relative to gender. Female *n* = 1403; male *n* = 1425. Sigmoidal (four parameter logistic) curves were fitted to the dose-concentration data with asymmetrical 95% confidence intervals indicated. Dashed line indicates 50 µmol/L serum vitamin C, which is considered ‘adequate’.

The vitamin C dose-concentration relationship between genders is shown in Figure 2. Females attained significantly higher serum vitamin C concentrations relative to intake compared with males. For females, a vitamin C intake of 72 (63, 84) mg/day was sufficient to reach the ‘adequate’ vitamin C concentration of 50 µmol/L, whereas males required a dietary intake of 127 (102, 174) mg/day to reach the same circulating concentration. Furthermore, males achieved a lower maximal plasma concentration of 53 (50, 56) µmol/L at an intake of 200 mg/day, relative to the 63 (60, 66) µmol/L attained by females at the same intake. The difference between the 95% CIs of the male and female subgroups was 18 mg/day, which corresponds to a 1.2-fold higher vitamin C requirement for males.

Smoking is well known to affect vitamin C status and requirements due to enhanced oxidative stress, which could be impacting on the male vitamin C requirements as the prevalence of smoking was significantly higher in males than females (29% vs. 19%, *p* < 0.0001). Higher body weight is also known to negatively impact vitamin C status and requirements; males had higher body weight than females (85 [73, 100] kg vs. 76 [63, 92] kg, *p* < 0.0001), which could also be negatively affecting the male vitamin C requirements. The impact of both smoking and body weight on vitamin C dose-concentration relationships are explored further below.

### 3.3. Vitamin C Dose-Concentration Relationship Relative to Smoking Status

Of the total cohort, 25% were smokers. The characteristics of the smokers relative to non-smokers are shown in Table 3. The proportion of males was significantly higher in the smoking group (61%). A higher proportion of smokers were non-Hispanic white and non-Hispanic black, and smokers also had a lower socioeconomic status. The body weights of the two groups were comparable, although the BMI of the smokers was slightly lower. Smokers had significantly lower vitamin C dietary intakes and circulating vitamin C concentrations than non-smokers (*p* < 0.0001). In those who smoked cigarettes (*n* = 567), the median number of cigarettes smoked per day was 8 (3, 15). Furthermore, linear regression indicated an inverse correlation between the number of cigarettes smoked per day and vitamin C status (r = −0.204, *p* < 0.0001). Based on this regression line, even people who had smoked the equivalent of only one cigarette in the previous five days had vitamin C levels well below adequate (37 [34, 40] µmol/L).

**Figure 3:**
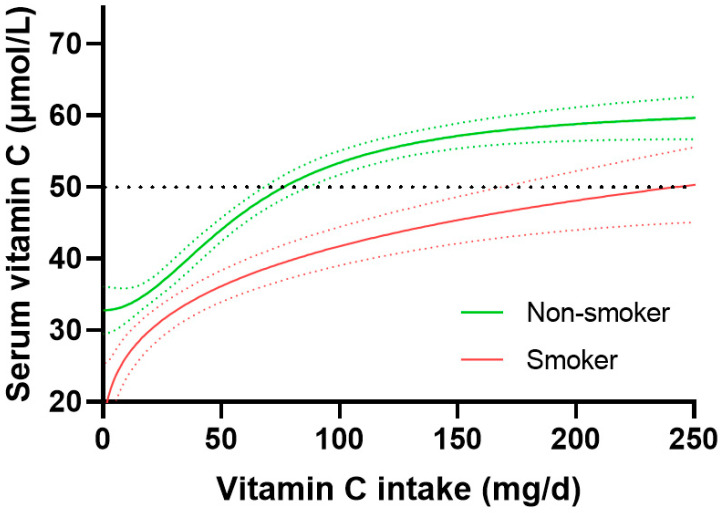
Vitamin C dose-response relationship relative to smoking status. Non-smoker *n* = 2068; smoker *n* = 681. Sigmoidal (four parameter logistic) curves were fitted to the dose-concentration data with asymmetrical 95% confidence intervals indicated. Dashed line indicates 50 µmol/L serum vitamin C, which is considered ‘adequate’.

Examination of the dose-concentration relationship stratified by smoking status confirmed a higher requirement of vitamin C for smokers to reach comparable serum concentrations to non-smokers (Figure 3). Non-smokers reached 50 µmol/L serum concentrations with intakes of 76 (67, 85) mg/day, whereas smokers required a much larger estimated intake of 236 (167, NA) mg/day to reach adequate plasma concentrations. Furthermore, non-smokers reached a maximum vitamin C concentration of 59 (56, 61) µmol/L with intakes of 200 mg/day, whereas smokers reached only 48 (45, 52) µmol/L serum concentrations with the same intake. The difference between the 95% CIs of the smoking and non-smoking groups was 82 mg/day, corresponding to a 2.0-fold increased requirement for vitamin C for smokers.

When smoking status was further stratified by gender, the difference in requirement between smokers and non-smokers persisted (Supplemental Figure S1). Female non-smokers required a dietary intake of 62 (52, 71) mg/d to reach 50 µmol/L serum concentrations and reached a maximal concentration of 65 (61, 67) µmol/L at an intake of 200 mg/day, versus female smokers who required a larger 166 (103, NA) mg/day vitamin C intake to reach 50 µmol/L, and attained 52 (42, 61) µmol/L circulating concentrations at an intake of 200 mg/day. Male non-smokers required 108 (87, 142) mg/day of vitamin C to reach a serum concentration of 50 µmol/L and attained concentrations of 54 (51, 57) µmol/L at intakes of 200 mg/day, whereas male smokers did not reach adequate vitamin C concentrations, attaining serum concentrations of only 44 (40, 48) µmol/L at intakes of 200 mg/day.

### 3.4. Vitamin C Dose-Concentration Relationship Relative to Body Weight

To determine the impact of body weight on the vitamin C dose response relationship, the cohort was stratified by weight tertile (<72 kg, 72–91 kg, >91 kg); the characteristics of the weight tertiles are shown in Table 4. There were significantly more females in the lower weight tertile (median female weight 76 [63, 92] kg) and more males in the higher weight tertile (median male weight 85 [73, 100] kg). Of note, serum vitamin C concentrations were significantly lower in the higher weight tertiles (*p* < 0.0001), despite a barely significant difference in vitamin C intake between the weight tertiles (*p* = 0.01). Linear regression showed an inverse correlation between body weight and vitamin C status (*r* = −0.198, *p* < 0.0001). Of note, linear regression indicated that only people who weighed 53 (45, 60) kg, or less, were able to reach adequate serum vitamin C concentrations of 50 µmol/L in this cohort.

**Figure 4:**
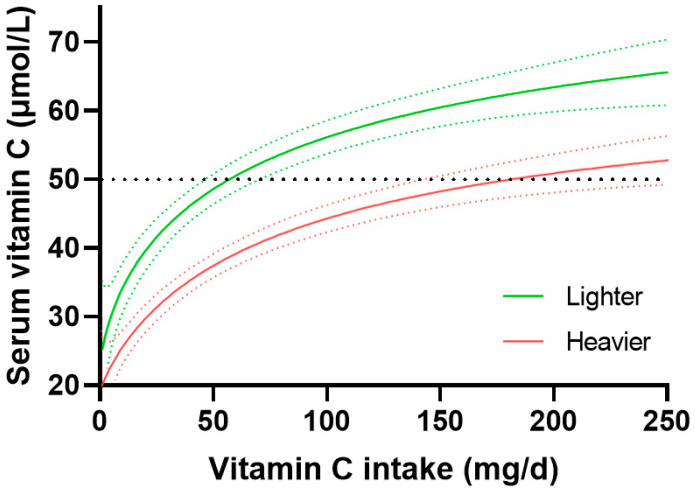
Vitamin C dose-response relationship relative to body weight. Lighter weight tertile 63 (57, 68) kg, *n* = 932; heavier weight tertile 105 (67, 118) kg, *n* = 930. Sigmoidal (four parameter logistic) curves were fitted to the dose-concentration data with asymmetrical 95% confidence intervals indicated. Dashed line indicates 50 µmol/L serum vitamin C, which is considered ‘adequate’.

The dose-concentration curves stratified by lighter and heavier weight tertiles are shown in Figure 4. The lighter group (63 [57, 68] kg) reached 50 µmol/L vitamin C concentrations at an intake of 56 (45, 70) mg/day, whereas the heavier group (105 [67, 118] kg) required 177 (140, NA) mg/day to reach the same concentration. Furthermore, the maximal doses attained at an intake of 200 mg/day were significantly different between the two groups; 51 (48, 54) µmol/L for the heavier tertile and 63 (60, 67) µmol/L for the lighter tertile. The middle-weight tertile (80 [76, 85] kg) was comparable to the total cohort dose-concentration relationship, with a dietary vitamin C intake of 90 (76, 111) mg/day required to reach a 50 µmol/L serum concentration, and attaining a maximum vitamin C concentration of 56 (52, 59) µmol/L at a dietary intake of 200 mg/day (Supplementary Figure S2). The difference between the 95% CIs for the lower and higher weight tertiles was 70 mg/day and corresponded to a 2.0-fold increased vitamin C requirement for the higher weight group. In order to compensate for the effect of smoking on the dose-concentration relationship, only non-smokers were analysed (*n* = 2052). Comparable trends were observed: the lighter non-smokers required 44 (32, 54) mg/day, the medium weight non-smokers required 76 (65, 95) mg/day and the heavier non-smokers required 144 (112, NA) mg/day of vitamin C to reach adequate serum concentrations. This equated to a difference between the upper and lower 95% CIs of the lighter and heaver weight groups of 57 mg/day, which corresponded to a 2.0-fold higher requirement for heavier non-smokers.

Because there were significant differences in the gender profiles of the higher and lower weight categories, the data was categorised by gender and the higher and lower weight tertile of each gender compared (Supplementary Figure S3). The weight-related difference in the dose-concentration relationships persisted, with the intakes required to reach 50 µmol/L in lighter (59 [34, 70] kg) and heaver (100 [92, 114] kg) females being 45 (32, 54) mg/day versus 116 (82, 154) mg/day, respectively, and lighter (69 [63, 73] kg) and heavier (108 [100, 120] kg) males being 71 (51, NA) mg/day versus 243 (172, NA) mg/day, respectively. At intakes of 200 mg/day, lighter females attained serum vitamin C concentrations of 67 (62, 72) µmol/L relative to 54 (48, 61) µmol/L for heavier females, and lighter males attained 53 (48, 58) µmol/L versus 48 (44, 52) µmol/L for heavier males. Comparable trends were observed when the dose-concentration data was investigated relative to BMI (Supplementary Figure S4) and stratified by gender (Supplementary Figure S5).

### 3.5. Vitamin C Dose-Concentration Relationship Relative to Age

We have recently published a report around the vitamin C dose-concentration relationship relative to age [12]. Relevant data is reproduced here for completeness and extended by also assessing the middle age group. The cohort was stratified by age tertiles: 18–36 y, 37–58 y, and 59–80+ y for further analyses (Table 5). There were fewer smokers in the older age group relative to the younger age group (18% vs. 27%, *p* < 0.0001) and a higher socioeconomic status in the older age group (*p* = 0.0007). Despite a comparable dietary intake between the age groups, the middle and older age groups had significantly lower serum vitamin C status than the younger age group (*p* < 0.0001). Linear regression indicated a weak but significant inverse association between age and vitamin C status (*r* = −0.086, *p* < 0.0001).

**Figure 5:**
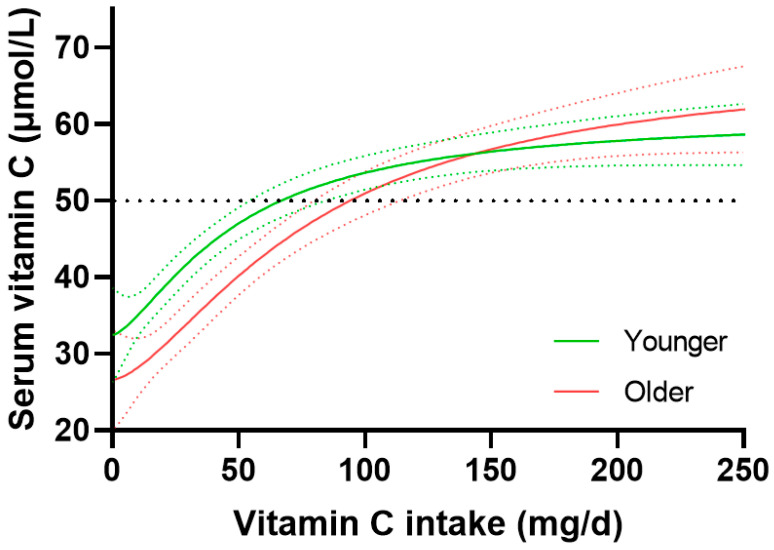
Vitamin C dose-response relationship relative to age. Younger age tertile 18–36 years (*n* = 942) vs. older age tertile 59–80+ years (*n* = 944). Sigmoidal (four parameter logistic) curves were fitted to the dose-concentration data with asymmetrical 95% confidence intervals indicated. Dashed line indicates 50 µmol/L serum vitamin C, which is considered ‘adequate’. Figure reproduced from [12].

Dose-concentration relationships in the younger versus older age groups indicated overlap between the upper and lower 95% CIs, respectively, at the intakes required to reach 50 µmol/L circulating concentrations (Figure 5), i.e., 66 (54, 83) mg/day for the younger group versus 95 (80, 112) mg/day for the older group. The maximal concentrations attained at an intake of 200 mg/day were also comparable between the young and old age groups (58 [55, 61] µmol/L versus 60 [56, 64] µmol/L, respectively). Of note, at intakes < 75 mg/day, there appeared to be an attenuated serum response to vitamin C intake in the older age group. The maximal difference between the 95% CIs of the younger and older age groups equated to ~10 mg/day. For the middle age cohort, the steady state concentrations attained at 200 mg/day did not differ to the younger or older age groups, i.e., 54 (51, 57) µmol/L, and although the dose-response relationship did not differ significantly from that of the older cohort, i.e., 126 (101, 160) mg/day to reach 50 µmol/L, this was significantly different to the younger age group (Supplementary Figure S6), the difference between the 95% CIs of the younger and middle age groups being 18 mg/day, corresponding to a 1.2-fold increased requirement.

### 3.6. Vitamin C Dose-Response Relationship Relative to Ethnicity

The cohort was stratified by ethnicity (Table 6): non-Hispanic white, non-Hispanic black and total Hispanic (comprising Mexican-American and other Hispanic). The non-Hispanic black group had a slightly higher weight/BMI (*p* < 0.01) and dietary intake (*p* = 0.002) than the non-Hispanic white group, but comparable smoking prevalence (*p* = 0.3) and circulating vitamin C status (*p* = 0.1). The Hispanic group had a much lower prevalence of smoking (*p* < 0.001) and a higher vitamin C dietary intake and circulating status (*p* < 0.001) than the other two ethnicities.

**Figure 6:**
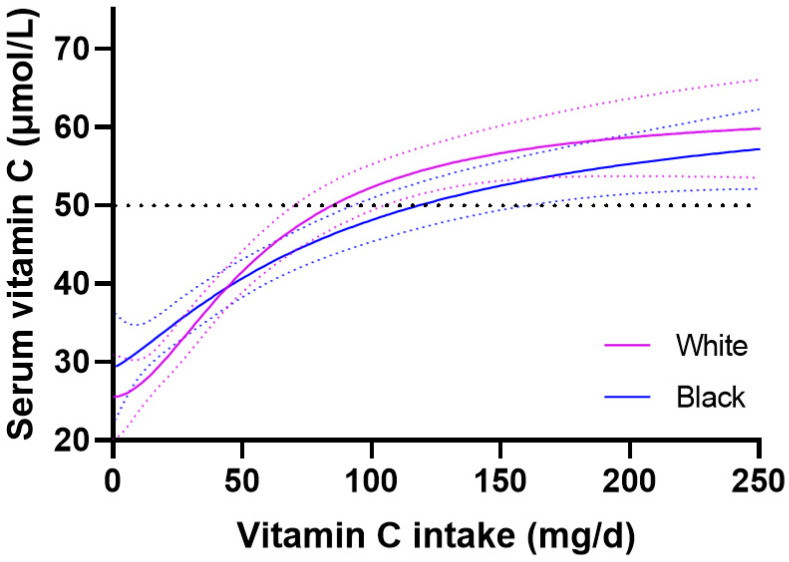
Vitamin C dose-response relationship relative to ethnicity. Non-Hispanic white (*n* = 940), and non-Hispanic black (*n* = 728). Sigmoidal (four parameter logistic) curves were fitted to the dose-concentration data with asymmetrical 95% confidence intervals indicated. Dashed line indicates 50 µmol/L serum vitamin C which is considered ‘adequate’.

An investigation of the dose-response relationships between non-Hispanic white and non-Hispanic black ethnicities indicated overlap in the 95% confidence intervals between the two groups (Figure 6). The non-Hispanic white group reached 50 µmol/L circulating concentrations with intakes of 86 (70, 105) mg/day versus 121 (92, 157) mg/day for non-Hispanic black group, and serum concentrations of 59 (54, 64) µmol/L versus 55 (51, 59) µmol/L for non-Hispanic blacks at steady-state intake of 200 mg/day. The Hispanic dose-response curve overlapped with these two groups, with an intake of 81 (62, 110) mg/day required to reach 50 µmol/L, and a maximal circulating concentration of 55 (51, 59) µmol/L at an intake of 200 mg/day (Supplementary Figure S7).

### 3.7. Vitamin C Dose-Response Relationship Relative to Socioeconomic Status

The socioeconomic status of the participants was assessed using the household income to poverty ratio (PIR), with higher values indicating higher socioeconomic status (range 0–5+). The lower PIR group was significantly younger and comprised a higher proportion of smokers (Table 7). There were also stark differences in the ethnic makeup of the PIR groups. Although there were no significant differences between the groups with respect to weight/BMI, the lower PIR group had a significantly lower vitamin C intake (*p* < 0.0001) and correspondingly lower serum vitamin C status (*p* = 0.0003). Linear regression indicated a weak positive correlation between PIR and vitamin C status (*r* = 0.085, *p* < 0.0001).

**Figure 7:**
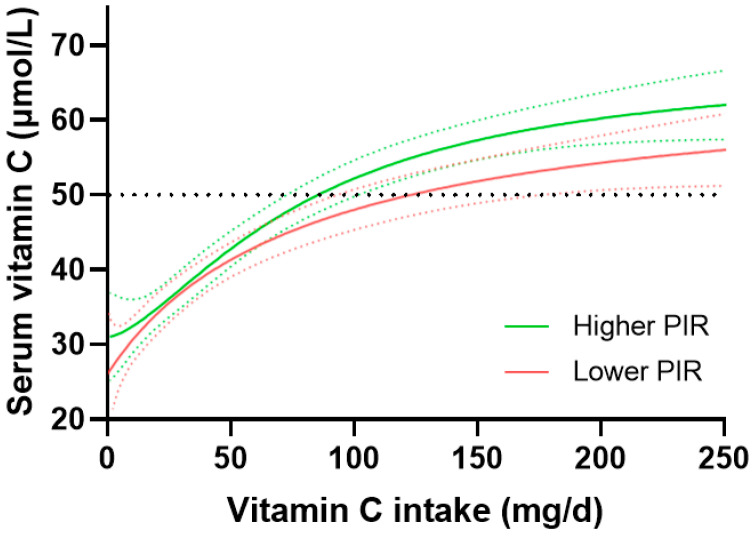
Vitamin C dose-response relationship relative to socioeconomic status. Higher income to poverty ratio tertile (PIR > 3.0, *n* = 843), and lower income to poverty ratio tertile (PIR ≤ 1.35, *n* = 843). Sigmoidal (four parameter logistic) curves were fitted to the dose-concentration data with asymmetrical 95% confidence intervals indicated. Dashed line indicates 50 µmol/L serum vitamin C, which is considered ‘adequate’. PIR, poverty income ratio.

Vitamin C dose-concentration data stratified by socioeconomic status is shown in Figure 7. The higher PIR group reached adequate vitamin C concentrations at an intake of 85 (73, 102) mg/day relative to the lower PIR group which required intakes of 123 (96, 174) mg/day to reach the same serum concentration, although this was not significantly different, as indicated by the overlap between the 95% CIs. At an intake of 200 mg/day, the higher PIR group attained serum concentrations of 60 (57, 63) µmol/L relative to 55 (51, 58) µmol/L for the lower PIR group, although once again this was not a statistically significant difference. The medium PIR group gave intermediate values: an intake of 94 (77, 122) mg/day was required to reach adequate serum status, and at 200 mg/day, the serum concentration attained was 57 (53, 61) µmol/L (Supplementary Figure S8).

## 4. Discussion

In this cohort of non-institutionalized and non-supplementing US adults, vitamin C dose-concentration relationships were examined to assess the impact of demographic and lifestyle factors (gender, smoking status, body weight, age, ethnicity and socioeconomic status) on these relationships. Although it is well established that males, smokers and heavier people tend to have lower vitamin C status, the impact of potential differences in dietary intakes on these findings is less clear. Therefore, assessing vitamin C status relative to dietary intake eliminates the impact of differences in dietary intake between subgroups. Using this methodology, we showed that males had higher vitamin C requirements than females, which was contributed to by their higher body weight and higher prevalence of smoking, as both smoking and body weight had a considerable impact on the vitamin C dose-concentration relationship (Table 8). Comparable trends were observed when the weight and smoking data was stratified by gender (Supplementary Table S1). In contrast, ethnicity and socioeconomic status did not impact significantly on the vitamin C dose-concentration relationship, whilst age had a variable impact depending on the age group.

### 4.1. Gender and Vitamin C Dose-Concentration Relationship

In agreement with other studies that have shown lower vitamin C status in males, despite comparable or higher dietary intakes than females [3], we confirmed that males had an attenuated serum response relative to vitamin C intake. In the total cohort, males required an additional vitamin C intake of ~18 mg/day relative to females to reach adequate serum concentrations (50 µmol/L), comprising a 1.2-fold enhanced requirement (Table 8). However, it is unlikely that it is gender, per se, that is impacting the dose-concentration relationship, but rather demographic and lifestyle differences between the genders, such as smoking and body weight. In well-controlled pharmacokinetic studies of healthy, non-smoking men and women, Levine and co-workers showed relatively comparable dose-concentration relationships between the two genders [5,6]. Although they observed a slightly higher plasma concentration in females at a comparable intake to males, the generally lower body weight of the healthy, non-smoking females (59 ± 9 kg) may have contributed to this.

### 4.2. Smoking and Vitamin C Dose-Concentration Relationship

Early radiolabel studies by Kallner and colleagues indicated higher turnover and requirements for vitamin C in smokers [17], and suggested that smokers should consume at least 140 mg/day of vitamin C relative to 100 mg/day in non-smokers. However, subsequent analysis of NHANES II (1976–1980) data by Schectman and colleagues indicated that smokers may have much higher vitamin C requirements than this, i.e., 233 mg/day relative to 100 mg/day for nonsmokers [25]. In their analysis, Schectman et al. fitted the dose-concentration data with linear regression lines, however, it is now known that the vitamin C dose-concentration relationship is non-linear in both men and women [5,6]. In a study with matched dietary intakes, smokers had significantly lower plasma concentration of vitamin C compared to non-smokers, but could be saturated with a supplement of 272 mg/day [18]. In our analysis of NHANES 2017–2018 data, we applied sigmoidal (four parameter logistic) curves to better fit the dose-concentration data. We showed that smokers required vitamin C intakes ~82 mg/day higher than non-smokers to reach adequate vitamin C concentrations, comprising a 2.0-fold higher requirement (Table 8). This is despite increased ascorbate recycling in smokers [26]. Thus, it appears that the controlled experiments of Kallner et al., suggesting that smokers should consume at least an additional 40 mg/day, do not translate to real-world populations, where smokers may need to consume an additional 82 mg/day of vitamin C relative to non-smokers. Of note, these were not necessarily heavy smokers as the median number of cigarettes smoked per day was less than 10 in the NHANES cohort. Stratification by gender indicated that female smokers required an additional 32 mg/day of vitamin C, a 1.5-fold enhanced requirement for the vitamin relative to female non-smokers (Supplementary Table S1), however, the smaller group sizes resulted in wider 95% CIs, thus this value is likely an underestimate. We were not able to calculate requirements for male smokers separately, due to the relevant 95% CIs not reaching the 50 µmol/L threshold.

### 4.3. Weight and Vitamin C Dose-Concentration Relationship

The dilution of vitamin C into larger volumes (as seen in the larger volume of distribution with increased weight) is of relevance to human health as the various enzymes relying on vitamin C as a cofactor require certain concentrations of the vitamin for optimal activity, which may not be met by lower concentrations of the vitamin resulting from a larger body [19]. Furthermore, the antioxidant activities of vitamin C are optimal at higher concentrations [27]. In the current analyses, relative to adults in the lighter weight tertile (<72 kg), adults in the heaver weight tertile (>91 kg) required an additional 70 mg/day of vitamin C to reach adequate serum concentrations, equating to a 2.0-fold enhanced requirement. Stratification by gender indicated that heavier females (>85 kg) required an additional 28 mg/day of vitamin C relative to lighter females (<67 kg) to reach adequate serum status, corresponding to a 1.5-fold enhanced requirement. We were not able to determine requirements for heavier males due to insufficient 95% CI data.

We have previously determined, using dose-concentration data from controlled studies of healthy non-smoking males [5,28], that an additional 1 mg of vitamin C is required for every additional kg of weight gain [13]. Thus, in comparison to a 60 kg person consuming ~100 mg/day of vitamin C, a 90 kg person would need to consume ~130 mg/day of the vitamin. In comparison, our ‘real-world’ data indicate that a higher ratio of vitamin C may be required, i.e., there was a ~29 kg difference in weight and a ~70 mg/day difference in intake between the upper and lower 95% CIs of the lighter and heavier weight tertiles, which equates to a requirement of ~2.4 mg vitamin C per kg of weight gained. For non-smokers, the requirement was ~2.2 mg vitamin C per kg weight gained. In addition to volumetric dilution, the larger vitamin C requirement at higher weights may be due to the enhanced inflammation and oxidative stress associated with obesity [29].

### 4.4. Age and Vitamin C Dose-Concentration Relationship

We recently reported that there was no significant difference between the dose-concentration data of younger and older adults at vitamin C intakes > 75 mg/day [12], which confirms earlier pharmacokinetic studies of both men and women carried out by Blanchard and coworkers [30,31]. However, we observed that at intakes < 75 mg/day, older adults had an attenuated serum response to vitamin C intake, requiring an additional ~10 mg/day to reach comparable serum status to younger adults. The middle age group had a similar profile to the older age group at lower intakes, and also had significantly higher requirements than the younger age group at higher intakes, i.e., requiring an additional 18 mg/day of vitamin C to reach 50 µmol/L serum concentrations, corresponding to a 1.2-fold increased requirement. The higher requirements of the middle and older age groups are likely contributed to by a higher prevalence of chronic health conditions and the effects of longer-term smoking on the body [12]. Although the impact of chronic health conditions was not assessed in this study, it is likely that these impact on vitamin C requirements and are of relevance to age-related requirements due to the continually growing aging population driving the global increase in chronic disease burden [32].

### 4.5. Ethnicity and Vitamin C Dose-Concentration Relationship

Varying vitamin C status in different ethnicities has been attributed to differing dietary, environmental and lifestyle factors [9]. Research has also indicated differences in sodium vitamin C transporter (SVCT) polymorphism profiles between white and black races, with African races having higher frequencies of the lower activity polymorphisms, which modelling has indicated may decrease the uptake of vitamin C from the diet resulting in lower circulating concentrations [33,34]. Nevertheless, in the NHANES cohort we did not observe any significant differences in the vitamin C dose-concentration relationship between black, white and Hispanic subgroups. The frequencies of detrimental polymorphisms may be too low in this cohort to have an impact on the observed dose-concentration relationships.

### 4.6. Socioeconomic Status and Vitamin C Dose-Concentration Relationship

Lower vitamin C concentrations are typically observed in people with lower socioeconomic status [35,36,37,38]. A major contributor to this is lower dietary intake of the vitamin [38], as foods of higher quality and nutritional value are generally out of reach of people with higher deprivation [39]. Smoking prevalence is also known to be higher among disadvantaged groups [40]. In our study, there was a non-significant trend towards higher vitamin C requirements in people of lower socioeconomic status; this was likely contributed to by the more than two-fold higher proportion of smokers in this group relative to the group with higher socioeconomic status (35% vs. 15%, respectively).

### 4.7. Study Limitations

The present approach, comparing tertiles of weight, BMI and age, etc., has limitations in the sense that these ranges may not compare completely to more physiologically based phases of life (e.g., fertility) or the established reference ranges (e.g., BMI) that could have been meaningful to investigate in relation to vitamin C. However, as a more physiologically or reference interval-based subdivision of the cohort would necessarily lower the power of the statistical analysis, we chose to maintain our approach throughout the study for consistency, and to optimize the applicability of the findings. Nevertheless, the values derived from the 95% CIs are still conservative estimates, being dependent on the sample size, thus, these could be further improved with larger numbers. Furthermore, the findings reported are specific to the cohort described; other cohorts with different characteristics may provide different absolute values, although the observed trends will likely remain. Finally, it is important to stress that the present study is purely observational and does not have the ability to establish causality. Thus, the factors identified in this study as the major determinants of the dose versus concentration relationship for vitamin C in humans may, in principle, be confounded by other, as yet unknown, correlating factors.

### 4.8. Implications for Global Vitamin C Dietary Recommendations

Our findings have important implications for the setting of vitamin C dietary recommendations. The European Food Safety Authority (EFSA) in 2013 first proposed the use of 50 µmol/L plasma vitamin C as indicating ‘adequate’ status and provided a strong rationale for this approach based on well-established pharmacokinetic principals [4]. In the current cohort of non-institutionalized, non-supplementing general public participants with a median weight of 80 (68, 97) kg, a vitamin C intake of 93 (83, 107) mg/day was required to reach 50 µmol/L circulating concentrations. Since the upper 95% CI is close to 110 mg/day, our data from a general public cohort support the recommendations of EFSA and the French, German, Austrian and Swiss health authorities, which have recommended vitamin C intakes of 110 mg/day for both men and women, or for men only [19]. The Italian, Singaporean, Japanese and Chinese health authorities have recommendations close to this (i.e., 105 mg/day for men or 100 mg/day for both men and women) [19]. However, there remain many countries and health authorities (e.g., WHO/FAO, UK, NZ/AUS) with vitamin C recommendations well below these intakes.

Gender differences have been taken into consideration by a number of health authorities, with lower vitamin C recommendations for women, based primarily on lower body weight [19]. Furthermore, the lower dietary recommendations for children and adolescents are also generally extrapolated from those of adults and are based on their lower body weight. Due to the considerable impact that body weight has on the vitamin C dose-concentration relationship, and the growing prevalence of overweight and obesity worldwide [21], we propose the introduction of weight-based recommendations or, at the very least, the introduction of a higher weight category (e.g., 100+ kg) with appropriately higher vitamin C intake recommendations (i.e., +2.2 mg vitamin C per kg weight gain over standard adult weight for non-smokers). This will help compensate for both volumetric dilution and the burden of obesity-related inflammation and oxidative stress.

The increased impact of smoking on vitamin C requirements has been considered by less than a handful of authorities, with additional vitamin C intakes of 20, 35, 45 or 80 mg/day recommended in France, the USA/Canada, Germany/Austria/Switzerland, and the UK, respectively [20,41,42,43]. As we and others have shown, additional intakes of <80 mg/day are likely to be insufficient for the additional demand that smoking places on vitamin C homeostasis. Furthermore, although there has been a general trend towards a decrease in smoking prevalence worldwide, some countries continue to show an increased trend in smoking [44]. As such, vitamin C recommendations of at least an additional 80 mg/day are indicated for smokers to help combat the significantly enhanced requirements for vitamin C. Female smokers may need less due to their lower body weight, i.e., closer to the current IOM recommendations for smokers [42].

With regard to vitamin C dietary recommendations for older people, to date, France is the only country with a higher recommended intake for adults aged 75 years and older (+10 mg/day), based on considerations related to immunity, cardiovascular risk, cancer risk, and cognition [4,20]. Our findings suggest that at vitamin C intakes < 75 mg/day, both middle age and older people have a lower serum response relative to vitamin C intake, requiring an additional 10–18 mg/day relative to younger people. This may have implications for those authorities with low vitamin C recommendations for adults, including the United Kingdom (40 mg/day) and WHO/FAO and Australia/New Zealand (45 mg/day) [19], recommendations which were originally intended to prevent scurvy. Even when following the recommendations of these health authorities, middle age and older people may have increased risk of experiencing lower plasma concentrations compared to younger adults with similar intakes. Ideally, the low recommendation countries should increase their RDAs for vitamin C to at least 75 mg/day to ensure all age groups within the population are adequately catered for.

## 5. Conclusions

In conclusion, specific demographic and lifestyle factors have significant impact on the vitamin C requirements necessary to achieve adequate serum concentrations. It is noteworthy that increased body weight has an equivalent impact on vitamin C requirements to smoking. The relatively smaller impacts of gender and age are confounded by weight and smoking differences between the subgroups. These findings have important implications for global vitamin C dietary recommendations and the alignment of these recommendations with specific population subgroups. Based on the data from the NHANES 2017–2018 cohort, our findings indicate that the general population should ideally consume approximately 110 mg/day to reach adequate circulating vitamin C concentrations of 50 µmol/L, and although males have higher requirements than females, the gender association can instead be accommodated by recommendations based on weight and smoking categories. Smokers require an additional intake of ~80 mg/day relative to non-smokers (i.e., a total of ~165 mg/day) and higher body weight requires an additional ~2.2 mg/kg over the average body weight of 80 kg, which equates to an additional ~45 mg/day for a 100+ kg weight category (i.e., ~155 mg/day total intake for heavier non-smokers). Tailoring vitamin C recommendations to the pertinent subgroups will help reduce inadequacy in these vulnerable groups within the population.

## Figures and Tables

**Table 1:** Cohort characteristics.

Characteristics	Total Cohort (*n* = 2828)
Age, years	48 (32, 62)
Sex, *n* (%):	
Male	1425 (50)
Female	1402 (50)
Ethnicity:	
Non-Hispanic white	940 (33)
Non-Hispanic black	728 (26)
Mexican American	399 (14)
Non-Hispanic Asian	328 (12)
Other Hispanic	281 (10)
Other/multi-race	152 (5)
Income to poverty ratio ^1^	2.0 (1.1, 3.8)
Current smoker	681 (25)
Body weight, kg	80 (68, 97)
Body Mass Index, kg/m^2^	29 (25, 34)
Vitamin C intake, mg/d	53 (24, 102)
Serum vitamin C, µmol/L	43 (23, 60)

Data represent median (Q1, Q3) or *n* (%). ^1^ Data were missing for income to poverty ratio for 302 (11%) participants, smoking status for 79 (2.8%) participants, body weight for 36 (0.9%) participants and BMI for 39 (1.0%) participants.

**Table 2:** Cohort characteristics stratified by gender.

Characteristics	Males (*n* = 1425)	Females (*n* = 1403)	*p* Value
Age, years	48 (32, 63)	47 (32, 61)	0.3
Sex, *n* (%):			
Male	1425 (100)	0 (0)	
Female	0 (0)	1403 (100)	<0.0001
Ethnicity:			
Non-Hispanic white	490 (34)	450 (32)	
Non-Hispanic black	354 (25)	374 (27)	
Mexican American	189 (13)	209 (15)	
Non-Hispanic Asian	168 (12)	160 (11)	
Other Hispanic	141 (10)	141 (10)	
Other/multi-race	83 (6)	69 (5)	0.5
Income to poverty ratio	2.0 (1.1, 3.9)	1.9 (1.0, 3.7)	0.06
Current smoker	413 (29)	268 (19)	<0.0001
Body weight, kg	85 (73, 100)	76 (63, 92)	<0.0001
Body Mass Index, kg/m^2^	28 (25, 33)	30 (25,36)	<0.0001
Vitamin C intake, mg/d	55 (24, 107)	52 (25, 97)	0.1
Serum vitamin C, µmol/L	39 (21, 55)	47 (27, 64)	<0.0001

Data represent median (Q1, Q3) or *n* (%).

**Table 3:** Cohort characteristics stratified by smoking status.

Characteristics	Non-Smokers (*n* = 2068)	Smokers (*n* = 681)	*p* Value
Age, years	49 (32, 63)	43 (31, 58)	<0.0001
Sex, *n* (%):			
Male	980 (47)	413 (61)	
Female	1080 (53)	268 (39)	<0.0001
Ethnicity:			
Non-Hispanic white	654 (32)	275 (40)	
Non-Hispanic black	472 (23)	232 (34)	
Mexican American	336 (16)	51 (7)	
Non-Hispanic Asian	278 (13)	31 (5)	
Other Hispanic	229 (11)	39 (6)	
Other/multi-race	53 (8)	99 (5)	<0.0001
Income to poverty ratio	2.2 (1.2, 4.2)	1.5 (0.8, 2.5)	<0.0001
Current smoker	0 (0)	681 (100)	<0.0001
Body weight, kg	80 (68, 96)	81 (69, 98)	0.3
Body Mass Index, kg/m^2^	29 (25, 34)	28 (24, 34)	0.001
Vitamin C intake, mg/d	58 (27, 106)	41 (18, 84)	<0.0001
Serum vitamin C, µmol/L	45 (28, 62)	30 (13, 53)	<0.0001

Data represent median (Q1, Q3) or *n* (%).

**Table 4:** Cohort characteristics stratified by body weight tertiles.

Characteristics	Lighter Tertile (*n* = 932)	Middle Tertile (*n* = 943)	Heavier Tertile (*n* = 930)	*p* Value
Age, years	46 (28, 63)	50 (34, 62)	47 (33, 61)	0.006
Sex, *n* (%):				
Male	322 (35)	531 (56)	560 (60)	
Female	610 (65)	410 (43)	370 (40)	<0.0001
Ethnicity:				
Non-Hispanic white	296 (32)	276 (29)	362 (39)	
Non-Hispanic black	188 (20)	240 (25)	295 (32)	
Mexican American	113 (12)	161 (17)	121 (13)	
Non-Hispanic Asian	193 (21)	104 (11)	30 (3)	
Other Hispanic	111 (12)	107 (11)	60 (6)	
Other/multi-race	31 (3)	55 (6)	62 (7)	0.04
Income to poverty ratio	2.0 (1.1, 3.9)	2.0 (1.1, 3.9)	2.0 (1.1, 3.7)	0.7
Current smoker	212 (23)	226 (24)	238 (26)	0.5
Body weight, kg	63 (57, 68)	80 (76, 85)	105 (97, 118)	<0.0001
Body Mass Index, kg/m^2^	24 (21, 26)	29 (27, 32)	36 (33, 41)	<0.0001
Vitamin C intake, mg/d	56 (28, 106)	54 (25, 100)	49 (21, 97)	0.01
Serum vitamin C, µmol/L	50 (28, 67)	44 (26, 59)	36 (19, 53)	<0.0001

Data represent median (Q1, Q3) or *n* (%).

**Table 5:** Cohort characteristics stratified by age.

Characteristics	Younger Tertile (*n* = 942)	Middle Tertile (*n* = 942)	Older Tertile (*n* = 944)	*p* Value
Age, years	26 (25, 32)	48 (42, 53)	66 (62, 73)	<0.0001
Sex, *n* (%):				
Male	475 (50)	452 (48)	498 (53)	
Female	467 (50)	490 (52)	446 (47)	0.1
Ethnicity:				
Non-Hispanic white	298 (32)	281 (30)	361 (38)	
Non-Hispanic black	225 (24)	235 (25)	268 (28)	
Mexican American	143 (15)	149 (16)	107 (11)	
Non-Hispanic Asian	130 (14)	133 (14)	65 (7)	
Other Hispanic	85 (9)	89 (9)	107 (11)	
Other/multi-race	61 (6)	55 (6)	36 (4)	0.06
Income to poverty ratio	1.8 (1.0, 3.4)	2.0 (1.1, 4.1)	2.1 (1.2, 3.8)	0.0007
Current smoker	246 (27)	269 (29)	166 (18)	<0.0001
Body weight, kg	79 (66, 96)	83 (70, 100)	80 (69, 95)	<0.0001
BMI, kg/m^3^	28 (23, 34)	30 (26, 35)	29 (26,34)	<0.0001
Vitamin C intake, mg/d	50 (22, 96)	54 (25, 106)	55 (27, 103)	0.07
Serum vitamin C, µmol/L	48 (29, 63)	40 (21, 57)	41 (21, 49)	<0.0001

Data represent median (Q1, Q3) or *n* (%).

**Table 6:** Cohort characteristics stratified by ethnicity.

Characteristics	Non-Hispanic White (*n* = 940)	Total Hispanic ^1^ (*n* = 680)	Non-Hispanic Black (*n* = 728)	*p* Value
Age, years	49 (33, 66)	47 (31, 61)	51 (32, 62)	0.002
Sex, *n* (%):				
Male	490 (52)	330 (49)	352 (49)	
Female	450 (48)	350 (51)	374 (51)	0.2
Ethnicity:				
Non-Hispanic white	940 (100)	0 (0)	0 (0)	
Non-Hispanic black	0 (0)	0 (0)	728 (100)	
Total Hispanic	0 (0)	680 (100)	0 (0)	<0.0001
Income to poverty ratio	2.1 (1.2, 4.2)	1.6 (0.9, 2.9)	1.7 (1.0, 3.3)	<0.0001
Current smoker	275 (30)	90 (14)	232 (33)	<0.0001
Body weight, kg	83 (69, 100)	80 (69, 92)	85 (72, 103)	<0.0001
Body Mass Index, kg/m^2^	29 (25, 35)	30 (27, 34)	30 (25, 36)	0.005
Vitamin C intake, mg/d	42 (21, 86)	64 (31, 117)	51 (23, 99)	<0.0001
Serum vitamin C, µmol/L	36 (17, 60)	46 (32, 63)	42 (22, 57)	<0.0001

Data represent median (Q1, Q3) or *n* (%). ^1^ Total Hispanic comprises Mexican American (*n* = 399) and Other Hispanic (*n* = 281).

**Table 7:** Cohort characteristics stratified by socioeconomic status.

Characteristics	Higher PIR (*n* = 840)	Medium PIR (*n* = 843)	Lower PIR (*n* = 843)	*p* Value
Age, years	49 (34, 62)	49 (32, 64)	44 (29, 60)	<0.0001
Sex, *n* (%):	432 (51)			
Male	408 (49)	440 (52)	398 (47)	
Female	445 (53)	350 (51)	445 (53)	0.09
Ethnicity:				
Non-Hispanic white	317 (38)	311 (37)	259 (31)	
Non-Hispanic black	175 (21)	199 (24)	245 (29)	
Mexican Hispanic	79 (9)	128 (15)	137 (16)	
Non-Hispanic Asian	174 (21)	79 (9)	45 (5)	
Other Hispanic	59 (7)	77 (9)	102 (12)	
Other/multi-race	36 (4)	49 (6)	55 (7)	<0.0001
Income to poverty ratio ^1^	4.8 (3.8, 5.0)	2.0 (1.7, 2.4)	0.9 (0.6, 1.1)	<0.0001
Current smoker	120 (15)	194 (23)	286 (35)	<0.0001
Body weight, kg	80 (69, 96)	82 (69, 97)	80 (67, 97)	0.4
Body Mass Index, kg/m^2^	28 (25, 33)	29 (25, 35)	29 (24, 35)	0.08
Vitamin C intake, mg/d	61 (29, 111)	53 (25, 95)	49 (20, 100)	<0.0001
Serum vitamin C, µmol/L	45 (28, 61)	43 (23, 60)	40 (21, 58)	0.0003

Data represent median (Q1, Q3) or *n* (%). ^1^ Income to poverty ratio data was missing from 302 (11%) of the cohort.

**Table 8:** Summary of vitamin C requirements from dose-concentration curves.

Factor	Curve 1 Intake (mg/Day) ^1^	Curve 2 Intake (mg/Day)	∆ 95% CI Intake (mg/Day) ^2^	Increased Requirement (Fold)
Total cohort	93 (83, 107)			
Females vs. males	72 (63, 84)	127 (102, 174)	18	1.2
Smoking status:				
Non-smokers vs. smokers	76 (67, 85)	236 (167, NA)	82	2.0
Weight tertiles: ^2^				
Lighter vs. heavier	56 (45, 70)	177 (140, NA)	70	2.0

^1^ Estimated doses of vitamin C required to reach 50 µmol/L serum vitamin C concentrations. ^2^ Difference between the upper 95% CI of Curve 1 and the lower 95% CI of Curve 2. Total cohort *n* = 2828; females *n* = 1403; males *n* = 1425; non-smokers *n* = 2068; smokers *n* = 681; lighter (≤72 kg) *n* = 932; heavier (≥91 kg) *n* = 930. Data were estimated from sigmoidal (four parameter logistic) curves with asymmetrical 95% confidence intervals fitted to dose-concentration data. NA, not attainable.

## Data Availability

Data are publicly available from the Centers for Disease Control and Prevention’s National Center for Health Statistics: https://www.cdc.gov/nchs/nhanes/index.htm, accessed on 10 November 2022.

## Supplementary Materials

The following supporting information can be downloaded at: https://www.mdpi.com/article/10.3390/nu15071657/s1, Figure S1. Vitamin C dose-response relationship relative to smoking status stratified by gender; Figure S2. Vitamin C dose-response relationship relative to body weight; Figure S3. Vitamin C dose-response relationship relative to body weight stratified by gender; Figure S4. Vitamin C dose-response relationship relative to BMI; Figure S5. Vitamin C dose-response relationship relative to BMI stratified by gender; Figure S6. Vitamin C dose-response relationship relative to age; Figure S7. Vitamin C dose-response relationship relative to ethnicity; Figure S8. Vitamin C dose-response relationship relative to socioeconomic status; Table S1. Summary of intake and concentration estimates from dose-concentration curves.
