# Supplementary material for: Factors Affecting the Vitamin C Dose-Concentration Relationship: Implications for Global Vitamin C Dietary Recommendations
Source: Nutrients. 2023 Mar 29;15(7):1657. doi: 10.3390/nu15071657 (PMC10096887; doi:10.3390/nu15071657)
Supplement: Supplementary file 1 [file nutrients-15-01657-s001.zip › nutrients-2276364-supplementary.pdf]

nutrients

(Supplemental Material)

Factors Affecting the Vitamin C Dose-Concentration Relationship: Implications for Global Vitamin C Dietary Recommendations

Anitra C. Carr [1,*] and Jens Lykkesfeldt [2]

[1] Nutrition in Medicine Research Group, Department of Pathology and Biomedical Science, University of Otago, Christchurch 8011, New Zealand
[2] Faculty of Health & Medical Sciences, University of Copenhagen, DK-1870 Frederiksberg C, Denmark
* Correspondence: anitra.carr@otago.ac.nz

3. Results

3.3. Vitamin C dose-concentration relationship relative to smoking status

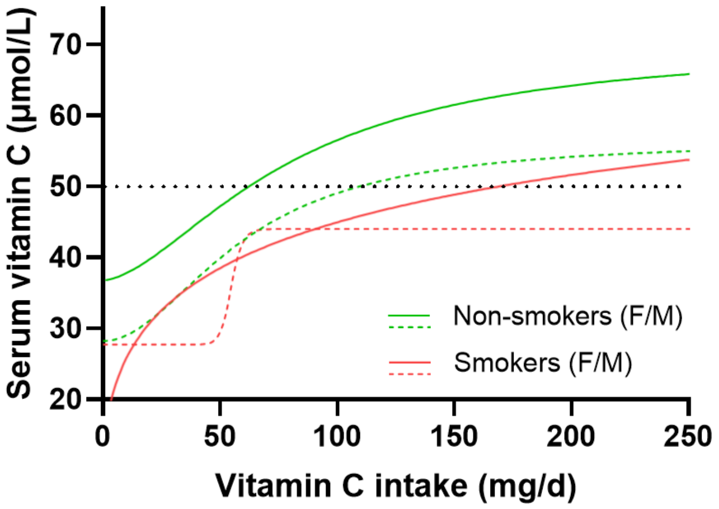

Figure S1. Vitamin C dose-response relationship relative to smoking status stratified by gender. Females are indicated by solid lines (non-smokers *n* = 1135, smokers *n* = 268); males are indicated by dashed lines (non-smokers *n* = 980, smokers *n* = 413). Sigmoidal (four parameter logistic) curves were fitted to the dose-concentration data. Dashed line indicates 50 µmol/L serum vitamin C which is considered 'adequate'. F, female; M, male.

3.4. Vitamin C dose-concentration relationship relative to body weight

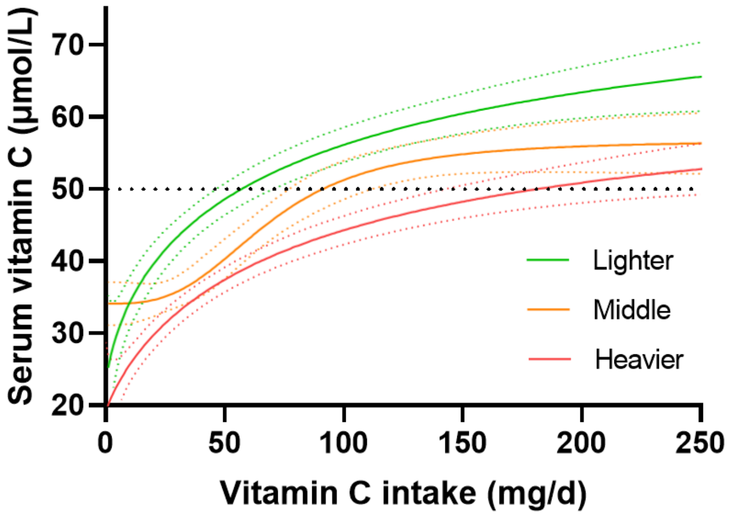

Figure S2. Vitamin C dose-response relationship relative to body weight. Lighter weight tertile 63 (57, 68) kg (*n* = 932); middle weight tertile 80 (76, 85) kg (*n* = 943); heavier weight tertile 105 (67, 118) kg (*n* = 930). Sigmoidal (four parameter logistic) curves were fitted to the dose-concentration data with asymmetrical 95% confidence intervals indicated. Dashed line indicates 50 µmol/L serum vitamin C which is considered 'adequate'.

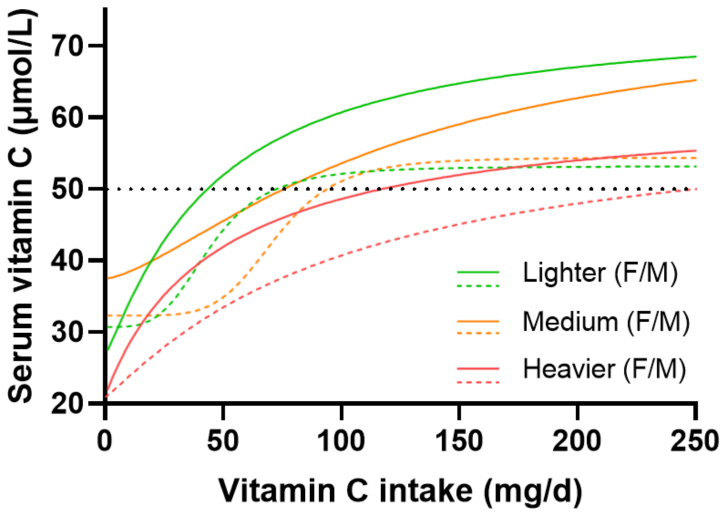

Figure S3. Vitamin C dose-response relationship relative to body weight stratified by gender. Female weight tertiles are indicated by solid lines (lighter 59 [34, 70] kg, *n* = 460; medium 75 [71, 80] kg, *n* = 471; heavier 100 [92, 114] kg, *n* = 461); male weight tertiles are indicated by dashed lines (lighter 69 [63, 73] kg, *n* = 469; medium 85 [80,90] kg, *n* = 474; heavier 108 [100, 120] kg, *n* = 470). Sigmoidal (four parameter logistic) curves were fitted to the dose-concentration data. Dashed line indicates 50 µmol/L serum vitamin C which is considered 'adequate'. F, female; M, male.

Vitamin C dose-concentration relationship relative to BMI

Linear regression indicated an inverse correlation between BMI and vitamin C status (r = -0.152, $p < 0.0001$). Of note, the upper 95% CI of the linear regression indicated that only people with BMIs of <20 kg/m^2 were able to reach adequate serum vitamin C concentrations of 50 µmol/L in this cohort. Dose-concentration analyses indicated that in the lower BMI category (23 [21, 25] kg/m^2) vitamin C intakes of 62 (51, 76) mg/day were required to reach 50 µmol/L circulating concentrations compared with intakes of 177 (140, NA) mg/day for the higher BMI category (37 [34, 41] kg/m^2; Figure S4a). Additionally, the lower BMI tertile attained a higher plasma concentration of 59 (55, 63) µmol/L at an intake of 200 mg/day versus a value of 51 (48, 54) µmol/L for the higher BMI tertile. The middle BMI category (29 [27, 32] kg/m^2) required an intake of 88 (76, 104) mg/day to reach 50 µmol/L circulating concentrations and attained 60 (57, 63) µmol/L serum concentrations at an intake of 200 mg/day (Figure S4b). The difference between the 95% CIs of the lower and higher BMI groups was 61 mg and corresponded to a 1.8-fold increased requirement for vitamin C in the higher BMI group.

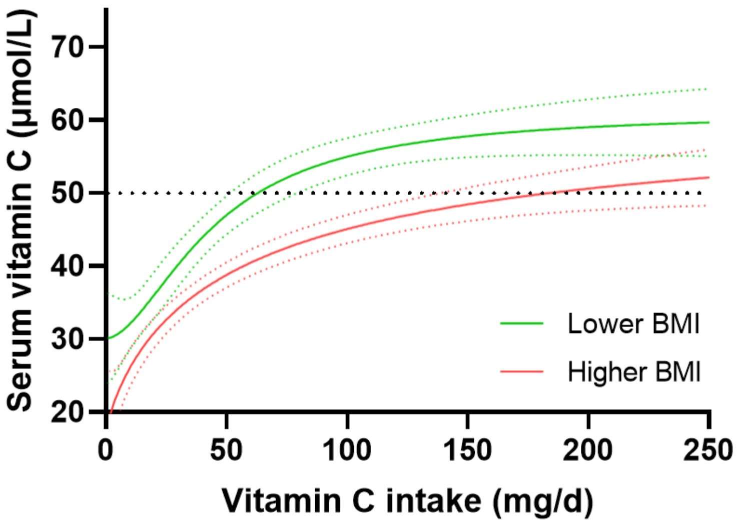

(**a**)

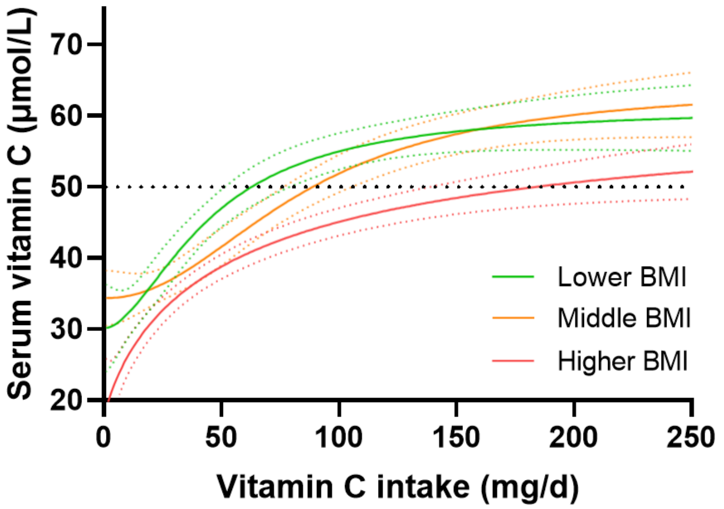

(**b**)

Figure S4. (a,b) Vitamin C dose-response relationship relative to BMI. Lower BMI tertile 23 (21, 25) kg/m^2 (n = 930); middle BMI tertile 29 (28, 31) kg/m^2 (n = 948); higher BMI tertile 37 (34, 41) kg/m^2 (n = 925). Sigmoidal (four parameter logistic) curves were fitted to the dose-concentration data with asymmetrical 95% confidence intervals indicated. Dashed line indicates 50 µmol/L serum vitamin C which is considered 'adequate'. BMI, body mass index.

Females of lower BMI (23 [21, 25] kg/m^2) required 43 (36, 54) mg/day to reach 50 µmol/L vitamin C concentrations and achieved 65 (58, 71) µmol/L at 200 mg/day intakes versus higher BMI females (39 [36, 43] kg/m^2) requiring 121 (90, NA) mg/day vitamin C to reach 50 µmol/L and achieving 54 (46, 62) µmol/L at an intake of 200 mg/day. Similarly, males of lower BMI (24 [22, 25] kg/m^2) reached 50 µmol/L vitamin C concentrations at an intake of 106 (69, NA) mg/day and achieved 53 (47, 60) µmol/L at an intake of 200 mg/day relative to males of higher BMI (35 [33, 38] kg/m^2) reaching 47 (43, 51) µmol/L concentrations at an intake of 200 mg/day.

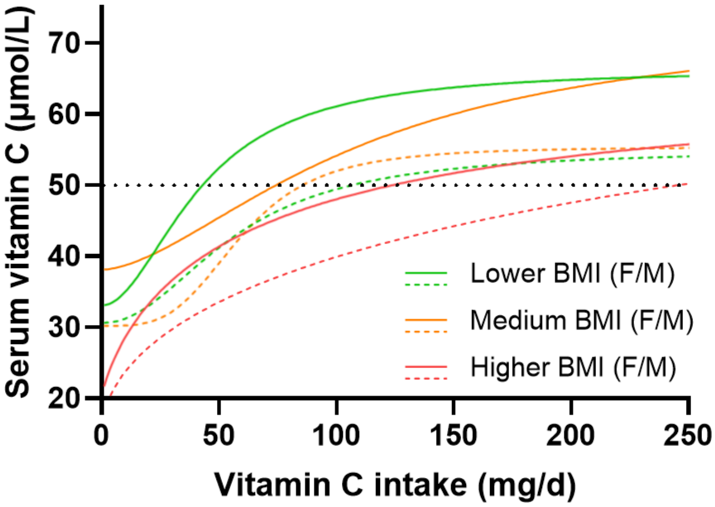

Figure S5. Vitamin C dose-response relationship relative to BMI stratified by gender. Female BMI tertiles are indicated by solid lines (lower 23 [21, 25] kg/m^2, *n* = 464; medium 30 [28, 31] kg/m^2 , n = 462; higher 39 [36, 43] kg/m^2, *n* = 466); male BMI tertiles are indicated by dashed lines (lower 24 [22, 25] kg/m^2, *n* = 469; medium 28 [27, 30] kg/m^2, *n* = 467; higher 35 [33, 38] kg/m^2, *n* = 475). Sigmoidal (four parameter logistic) curves were fitted to the dose-concentration. Dashed line indicates 50 µmol/L serum vitamin C which is considered 'adequate'. BMI, body mass index; F, female; M, male.

3.5. Vitamin C dose-concentration relationship relative to age

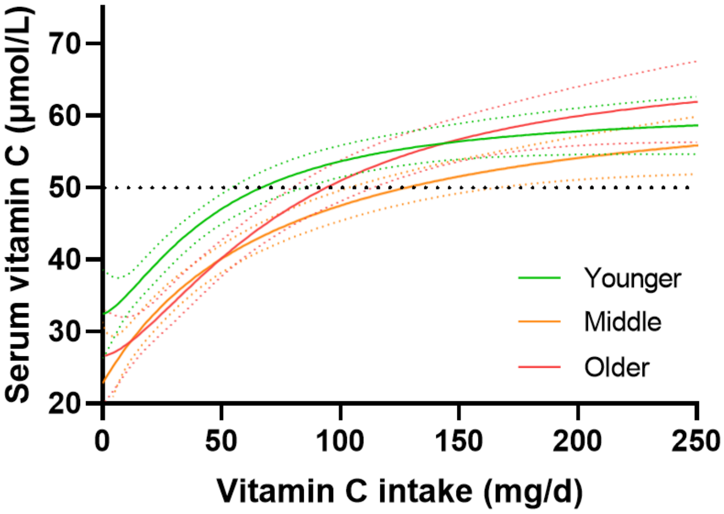

Figure S6. Vitamin C dose-response relationship relative to age. Younger age tertile 18-36 years (*n* = 942); middle age tertile 37-58 years (*n* = 942); older age tertile 59-80+ years (*n* = 944). Sigmoidal (four parameter logistic) curves were fitted to the dose-concentration data with asymmetrical 95% confidence intervals indicated. Dashed line indicates 50 µmol/L serum vitamin C which is considered 'adequate'.

3.6. Vitamin C dose-response relationship relative to ethnicity

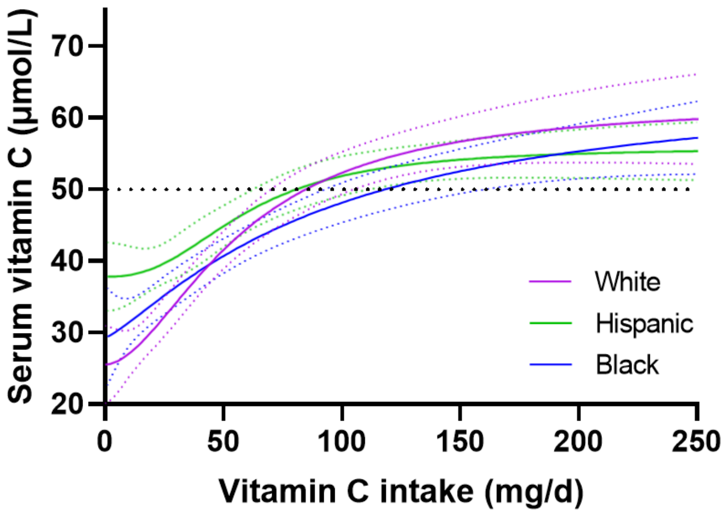

Figure S7. Vitamin C dose-response relationship relative to ethnicity. Non-Hispanic white (*n* = 940); total Hispanic (Mexican American and other Hispanic, *n* = 680); non-Hispanic black (*n* = 728). Sigmoidal (four parameter logistic) curves were fitted to the dose-concentration data with asymmetrical 95% confidence intervals indicated. Dashed line indicates 50 µmol/L serum vitamin C which is considered 'adequate'.

3.7. Vitamin C dose-response relationship relative to socioeconomic status

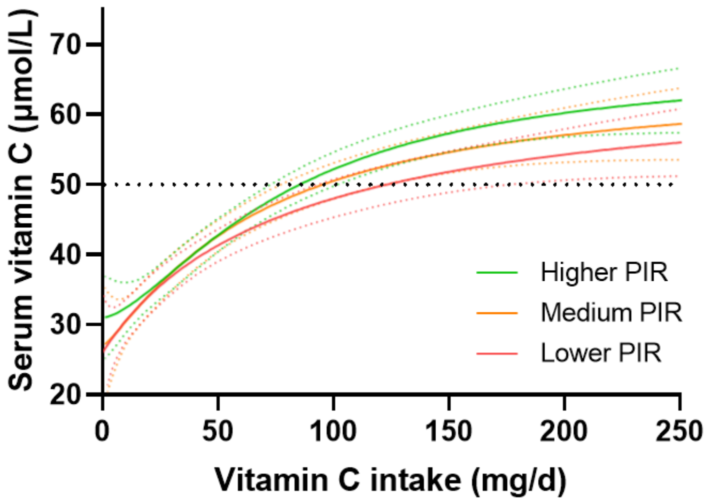

Figure S8. Vitamin C dose-response relationship relative to socioeconomic status. Higher poverty income ratio tertile (PIR >3.0, *n* = 840), medium poverty income ratio (PIR 1.36 – 3.0, *n* = 843), and lower poverty-income ratio tertile (PIR ≤1.35, *n* = 843). Sigmoidal (four parameter logistic) curves were fitted to the dose-concentration data with asymmetrical 95% confidence intervals indicated. Dashed line indicates 50 µmol/L serum vitamin C which is considered 'adequate'. PIR, poverty income ratio.

4. Discussion

Table S1. Summary of intake and concentration estimates from dose-concentration curves.

Factor	Total (n = 2828)		Females (n = 1403)		Males (n = 1425)	
	Dose (mg/day) [1]	Concentration (μmol/L) [2]	Dose (mg/day)	Concentration (μmol/L)	Dose (mg/day)	Concentration (μmol/L)
Total cohort	93 (83, 107)	57 (55, 59)	72 (63, 84)	63 (60, 66)	127 (102, 174)	53 (50, 56)
Smoking status:						
Non-smokers	76 (67, 85)	59 (56, 61)	62 (52, 71)	65 (61, 67)	108 (87, 142)	54 (51, 57)
Smokers	236 (167, NA)	48 (45, 52)	166 (103, NA)	52 (42, 61)	NA	44 (40, 48)
Weight tertiles: [3]						
Lighter	56 (45, 70)	63 (60, 67)	45 (32, 54)	67 (62, 72)	71 (51, NA)	53 (48, 58)
Middle	90 (76, 111)	56 (52, 59)	76 (57, 100)	63 (57, 68)	93 (79, 116)	54 (50, 59)
Heavier	177 (140, NA)	51 (48, 54)	116 (82, 154)	54 (48, 61)	243 (172, NA)	48 (44, 52)
BMI tertiles: [4]						
Lower	62 (51, 76)	59 (55, 63)	43 (36, 54)	65 (58, 71)	106 (69, NA)	53 (47, 60)
Middle	88 (76, 104)	60 (57, 63)	76 (58, 110)	64 (58, 69)	86 (68, 112)	55 (51, 59)
Higher	178 (137, NA)	51 (48, 54)	121 (90, NA)	54 (46, 62)	243 (185, NA)	47 (43, 51)

[1] Estimated doses of vitamin C required to reach 50 μmol/L serum vitamin C concentrations. [2] Estimated serum concentrations of vitamin C attained with an intake of 200 mg/day. [3] Weight tertiles for total cohort: lighter ≤72 kg; medium 72-91 kg; heavier ≥91 kg; Females: lighter ≤67 kg; medium 68-85 kg; heavier ≥85 kg; Males: lighter ≤77 kg; medium 77-94 kg; heavier ≥95 kg. [4] BMI tertiles for total cohort: lower ≤26 kg/m^2; medium 26-32 kg/m^2; higher ≥32 kg/m^2; Females: lower ≤26 kg/m^2; medium 26-33 kg/m^2; higher ≥33 kg/m2; Males: lower ≤26 kg/m^2; medium 26-31 kg/m^2; higher ≥31 kg/m^2. Data was estimated from sigmoidal (four parameter logistic) curves with asymmetrical 95% confidence intervals fitted to dose-concentration data. BMI, body mass index.
